# Hepatitis B and C Co-Infections in Some HIV-Positive Populations in Cameroon, West Central Africa: Analysis of Samples Collected Over More Than a Decade

**DOI:** 10.1371/journal.pone.0137375

**Published:** 2015-09-15

**Authors:** Jean Jacques N. Noubiap, Peter V. Aka, Aubin J. Nanfack, Lucy A. Agyingi, Johnson N. Ngai, Phillipe N. Nyambi

**Affiliations:** 1 Serology Unit, Medical Diagnostic Center, Yaounde, Cameroon; 2 Division of Cancer Epidemiology and Genetics, National Cancer Institute, Bethesda, Maryland, United States of America; 3 Department of Pathology, New York University School of Medicine, New York, New York, United States of America; 4 Faculty of Medicine and Surgery, Department of Immunology and Applied Biotechnology, University of Rome Tor Vergatta, Rome, Italy; 5 Faculty of Science, University of Dschang, Dschang, Cameroon; 6 Veterans Affairs New York Harbor Healthcare Systems, New York, New York, United States of America; CRCL-INSERM, FRANCE

## Abstract

As people infected with the human immunodeficiency virus (HIV) in Sub-Saharan Africa live longer due to availability of antiretroviral treatment (ART), so is the rise of associated infections with their burdens on patients. But reliable data on the prevalence of co-infection with hepatitis B (HBV) or C (HCV) still remains sparse and many individuals with HIV do not know their co-infection status. This study attempted to estimate the seroprevalence and identify risk factors associated with hepatitis B and/or C co-infections in HIV-infected individuals from five Regions of Cameroon by screening 531 HIV infected subjects for the presence of HBV surface antigen (HBsAg) and antibodies to HCV (HCV-Ab). A Screening and a confirmatory Enzyme linked immunosorbent assay were used to detect presence of markers of infection. CD4 count levels were also examined. The results indicate that of the 531 participants, 68% were females and 32% males. Mean CD4 count was ~400 cells/μl. Seroprevalence rates for HBsAg and HCV-Ab were 23.7%, and 7.2%, respectively. Associations assessed using logistic regression revealed that HBsAg but not HCV-Ab positivity was linked to age, lower CD4 count and residing in an urban rather than in a rural setting. This high prevalence of co-infection with HBV raises the urgent need to systematically screen all newly diagnosed HIV cases for co-infection in Cameroon and other regions of sub-Saharan Africa where HIV accounts for the majority of the global infection, so as to improve management strategies for HBV infection and ART implementation.

## Introduction

People infected with the human immunodeficiency virus (HIV) are at a greater risk of co-infection with either hepatitis B (HBV) and/or hepatitis C virus (HCV) compared to the general population [1]. Up to 33% of those with HIV may be co-infected with HBV or HCV [2]. These statistics are of particular importance in Sub-Saharan Africa where two thirds of the over 34 million people infected with HIV live (WHO, 2011). Co-infection by HBV or HCV in HIV infection is likely to result in chronic liver disease with potential for rapid progression to liver fibrosis, cirrhosis, end-stage liver disease, hepatocellular carcinoma (HCC) and mortality due to liver pathology [3]. HIV/HCV co-infected individuals are three times more likely to develop these complications than those with HIV infection alone [4].

Although the mechanisms by which the hepatitis virus interacts with HIV to influence disease progression are not well understood, it has been reported that HIV/HBV co-infection facilitates HBV replication and reactivation leading to higher HBV DNA levels and a reduced spontaneous clearance of the virus [5–8]. On the other hand, HCV may take advantage of the lowering of viral specific CD8+ T cell responses, chronic immune activation and increase in pro-inflammatory cytokines that follow infection by HIV to invade the host [4, 9, 10].

In most developing countries, including Cameroon, HCV and/or HBV testing and monitoring in HIV patients is not routine. As a result, the challenge of implementing directives from the World Health Organization (WHO) that ART be commenced in HIV co-infected patients irrespective of CD4 count results remains daunting. Therefore many HIV/hepatitis virus infected patients do not benefit from programs aimed at treating HIV patients since only the patients’ CD4 count level is taken into consideration for the purpose of initiating ART [11].

Worldwide an estimated 350 million people are chronically infected with HBV while 185 million are chronic carriers of HCV [12]. On the average ~15 and 7% of HIV-infected patients in sub-Saharan Africa are also infected with HBV or HCV, respectively [13, 14]. A systematic review of the epidemiology of HIV co-infection with HBV and HCV in sub-Saharan Africa reported an HBsAg prevalence of up to 20% in HIV infected patients in Cameroon [13]. A lower HBsAg prevalance of 10% was found amongst healthy blood donors attending a distric hospital [15]. Studies of other special groups put estimates of HCV prevalence in Cameroon at between 1–13% [15]. These data suggest that estimates of co-infection prevalence may vary depending on the risk groups and geographical area. The current study was therefore designed to estimate the prevalence of co-infection of HBV and/or HCV in people infected by HIV in Cameroon. Reliable epidemiological data is important in planning dedicated preventive health services aimed at this group of people.

## Materials and Methods

### Ethics Statement

Ethical approval for the current study was obtained from the National Ethical Review Board in Cameroon and the Institutional Review Board at the New York University School of Medicine. Patients’ records were anonymized and de-identified prior to analysis.

### Study population

All participants were initially enrolled as part of a large cohort study on HIV-1 genetic diversity in Cameroon [16–20]. All the study subjects were infected by HIV-1, ART naïve, and lived in rural villages or urban towns in the Centre, East, South, South-West, and North-West Regions of Cameroon. Thus, the samples used in this study are based on convenience sampling in that all the achieved plasma samples from the HIV-1 infected patients studied above for subtype diversity were analyzed for HBV and/or HCV co-infection. All the plasma samples used in this study were stored at -80°C in New York until testing. Because we had stored 5–10 aliquots of plasma samples from each patient, preferentially unthawed samples or aliquots of samples that were thawed only once were used in this study.

### Serology

#### Hepatitis B surface antigen

**Fig 1:**
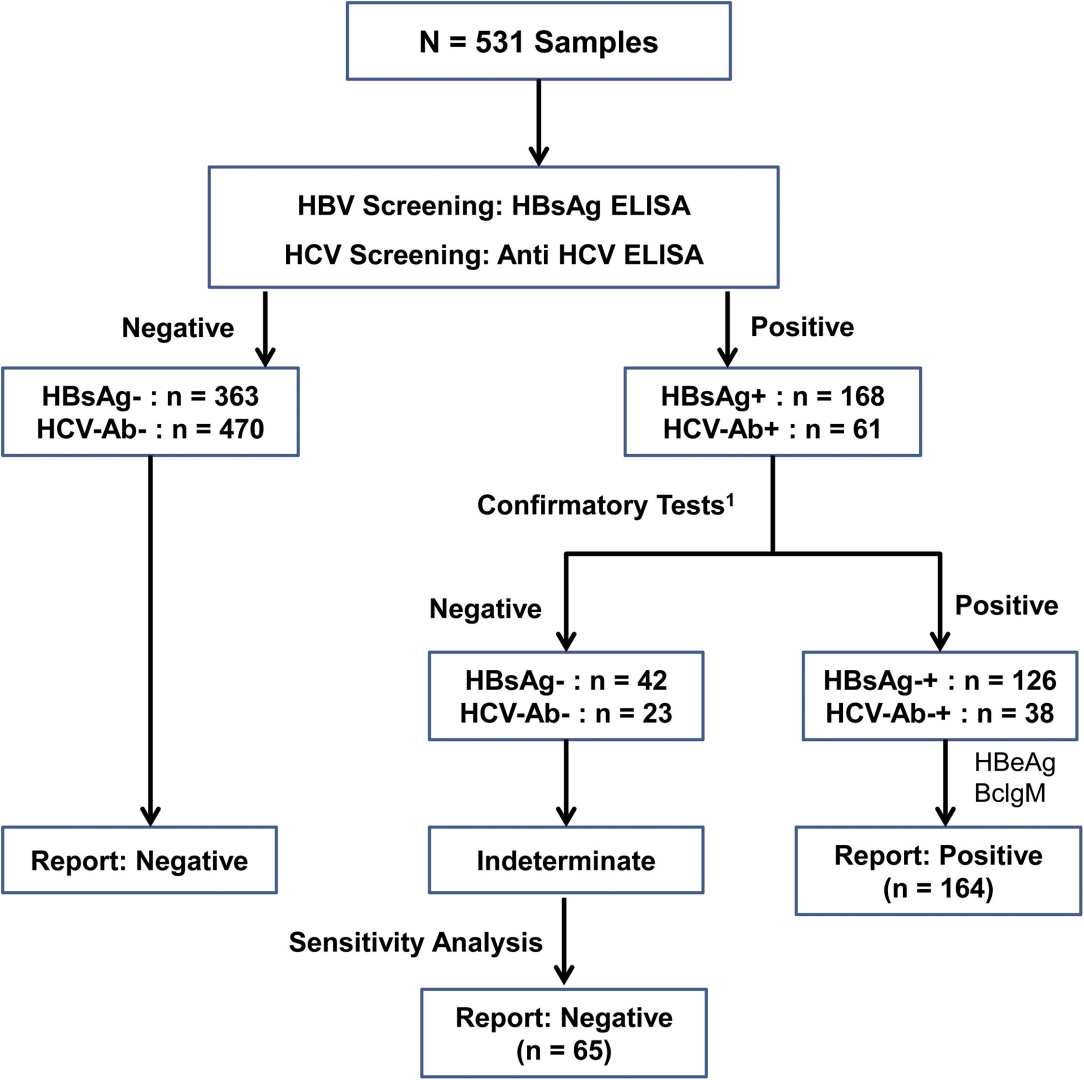
Viral testing algorithm. ^1^HBsAg: confirmatory test for HBsAg. Confirmatory test for HCV-Ab was the same test as for the screening for HCV-Ab.

Testing for the hepatitis B surface antigen (HBsAg) was performed using the HBsAg enzyme linked immunosorbent assay (ELISA) (DIAsource Immunoassay SA, Louvain-la-Neuve, Belgium) according to the manufacturer’s instructions. All non-reactive samples were recorded as negative. All reactive samples were tested a second time using the HBsAg confirmatory test according to the manufacturer’s instructions. All samples reactive to both assays were recorded as positive while those that were non-reactive in the second test after being reactive in the first test were considered non concordant and were subjected to a sensitivity analysis before being included as either positive or negative (Fig 1).

#### Hepatitis “e” antigen and IgM antibody to hepatitis B core antigen (HBc IgM)

All samples reactive by ELISA for HBsAg were further tested for the presence of hepatitis “e” antigen (HBeAg) and IgM antibody to hepatitis B core antigen (HBc IgM) using ELISA kits (DIAsource Immunoassay SA, Louvain-la-Neuve, Belgium).

#### Hepatitis C antibodies

The presence of hepatitis antibodies is a strong marker of infection with HCV. HCV-Ab was detected using the Anti-HCV ELISA V 4.0 (DIAsource Immunoassay SA, Louvain-la-Neuve, Belgium), a fourth generation enzyme immunoassay kit, which uses recombinant HCV antigens (Core, NS3 and NS5 antigens). HCV-Ab reactive samples were confirmed with the same ELISA kit using the algorithm shown in Fig 1. For HCV, all samples reactive to both the screening and confirmatory assays were recorded as positive while those that were non-reactive in the second test after being reactive in the first test were considered non-concordant and were subjected to a sensitivity analysis before being included as either positive or negative (Fig 1).

#### CD4 analysis

CD4 count of participants was determined by Guava Easy CD4 system that uses combination of monoclonal antibody and volumetric system to count cell numbers with high precision [21].

#### Statistical Evaluation of Data

Participants positive for HBsAg or anti-HCV following testing were considered infected. Continuous variables including age and CD4 counts were compared in males and females using the Student’s t-test. Categories were compared using the chi squared test for trend. Unconditional logistic regression was used to estimate odds ratios (ORs) and 95% confidence intervals (CIs) of the association between chronic infection with HBV or HCV and each of the defined risk factors including age, sex, CD4 count, residence, region, and number of sexual factors. We did not analyze by year of enrollment as participants in different regions were enrolled during different years. Each of the risk factors was entered into the regression model as a main effect and adjusted for the other co-factors including being infected with either HBV or HCV. P trend was estimated by treating categorized markers as ordinal variables. In a sensitivity analysis (not presented), inclusion of subjects with non-concordant results following testing with the second ELISA assay as non-reactive had no substantial effect on the results. Two-sided P values of <0.05 were considered statistically significant. All statistical analyses were performed using Stata (version 13, StataCorp, College Station, TX).

## Results

The total number of people initially screened for hepatitis viral infections were 531. Of these, 168 or 32% were positive for hepatitis B surface antigen (HBsAg+) while HCV antibodies (HCV-Ab) were detected in 61 or 11.5% (Table 1 and Fig 1). Nine or 2% tested positive for both HBsAg and HCV-Ab. Of the participants who tested positive in the screening tests, 126 (23.7%) and 38 (7.2%) also tested positive in confirmatory tests for HBsAg and HCV-Ab, respectively (Fig 1). Sixty two or about 12% of subjects did not have information on sex. Of the 469 with information on sex, 319 or 68% were female while 150 or 32% were male. Information on age was missing for 81 or 15% of participants. All participants were screened between 2000 and 2013, with about half screened during 2006 and 2010. The South West region had the highest percentage of those screened, (n = 204 or 38%), followed by the Center region (n = 181 or 34%), Table 1. Most of the people screened live in urban cities such as Yaounde, Limbe, etc, with only about 20% in rural villages.

**Table 1:** Demographic and serology characterization of participants.

**Characteristics**	Overall (%)	HBsAg+ (%)	HBeAg (%)	HCV Ab+ (%)
**All Subjects**	531 (100)	168 (31.6)	15 (2.8)	61 (11.5)
**Sex**				
Female	319 (60.1)	106 (63.1)	7 (46.7)	38 (63.3)
Male	150 (28.2)	40 (23.8)	5 (33.3)	17 (27.9)
Missing	62 (11.7)	22 (13.1)	3 (20.0)	6 (9.8)
**Age Groups**				
14–30	125 (23.5)	45 (26.8)	4 (26.7)	12 (19.7)
31–40	152 (28.6)	46 (27.3)	4 (26.7)	20 (32.8)
41–50	107 (20.2)	36 (21.4)	3 (20.0)	13 (21.3)
>50	66 (12.4)	14 (8.3)	0 (0.0)	10 (16.4)
Missing	81 (15.3)	27 (16.1)	4 (26.7)	6 (9.8)
**Year**				
2000–2004	131 (24.7)	28 (16.7)	7 (46.7)	19 (31.1)
2006–2010	256 (48.2)	55 (32.7)	4 (26.7)	17 (27.9)
2011–2013	144 (27.1)	85 (50.6)	4 (26.7)	25 (41.0)
**Region**				
Center	181 (34.1)	98 (58.3)	6 (40.0)	29 (47.5)
East	67 (12.6)	15 (8.9)	5 (33.3)	15 (24.5)
North West	67 (12.6)	23 (13.7)	1 (6.7)	6 (9.8)
South	12 (2.3)	1 (0.6)	0 (0.0)	0 (0.0)
South West	204 (38.4)	31 18.5)	3 (20.0)	11 (18.0)
**Residence**				
Rural	101 (19.0)	17 (10.1)	5 (33.3)	15 (24.6)
Urban	430 (81.0)	151 (89.9)	10 (66.7)	46 (75.4)
**Sexual Partners**				
One	182 (34.3)	54 (32.1)	4 (26.7)	22 (36.1)
Multiple	48 (9.0)	23 (13.7)	0 (0.0)	3 (4.9)
Missing	301 (56.7)	91 (54.2)	11 (73.3)	36 (59.0)
**CD4 status**				
<250cells/μl	65 (23.5)	26 (24.3)	2 (40.0)	6 (17.7)
≥250 cells/μl	212 (76.5)	81 (75.7)	3 (60.0)	28 (82.3)

Percentages (%) in columns were calculated by dividing the number of participants with a particular outcome by the total number of participants with that outcome of interest *100. For example, of all those with a positive result for HBsAg (HBsAg+), 63.1% were females. Column % adds-up to 100. HBsAg, Hepatitis B surface antigen, HCV-Ab, Antibodies to hepatitis C, HBeAg, Hepatitis B e antigen.

Table 2 outlines the baseline characteristics of the population analyzed in this study by sex. For participants with complete information on age and sex (n = 447), 299 or 67% were females while males were 148 or 33%. The males were on the average older than the females (mean age 41 vs 37, p = 0.003). CD4 counts did not differ between males (426.4±30.5 cells/μl) and females (400±15.7 cells/μl), p = 0.413. Of the 126 confirmed HBsAg+ patients, 32 or 25% were male while 79 or 63% were female, Ptrend = 0.415. Amongst those who were HBsAg+, 15 or 12% also tested positive for the hepatitis B e antigen (HBeAg). HBeAg positivity is an indication that viral replication is active. Therefore HBV replicative capacity did not differ between men and women, P = 0.247. HCV-Abs were detected in 38 or 7.2% of study participants. Although more female participants (n = 21 or 69%) tested HCV Ab+ than men (n = 13 or 38%) the trend was not statistically significant, P = 0.509. Only six participants or 1% were confirmed infected with both HBV and HCV (Table 2).

**Table 2:** Baseline characterization of HBV and HCV markers by sex.

Parameter/Infection Type	Baseline Characteristic			
	N (%)	Male	Female	P-value
**Age (mean ± SE)**	447 (100)	148 (41.15±1.1)	299 (37.48±0.65)	**0.003**
**Mean CD4 Count**	277 (62)	67 (427.0±30.1)	210 (400±15.4)	**0.413**
**HBsAg**				**0.715**
Positive	126 (23.7)	32 (21.3)	79 (24.8)	
Negative	405 (76.3)	118 (78.7)	240 (75.2)	
**HBeAg**				**0.247**
Positive	15 (8.9)	5 (12.5)	7 (6.6)	
Negative	153 (91.1)	35 (87.5)	99 (93.4)	
**HCV Ab**				**0.417**
Positive	38 (7.2)	13 (8.7)	21 (6.6)	
Negative	493 (92.8)	137 (91.3)	298 (93.4)	
**HBsAg and HCV Ab**				**0.183**
HBsAg-, HCV Ab-	323	99 (72.7)	188 (69.3)	
HBsAg+, HCV Ab+	6	4 (2.7)	2 (0.6)	
HBsAg+, HCV Ab-	113	28 (18.7)	71 (24.1)	
HBsAg-, HCV Ab+	28	8 (6.0)	17 (6.0)	

P-values for age and CD4 cell counts represent differences in mean between males and females. Other P-values stand for trend across categories of infection type. For viral markers of infection, % column was calculated as in Table 1.

SE, Standard error of the mean; HBsAg, Hepatitis B surface antigen; HBeAg, Hepatitis B e antigen; HCV-Ab, Antibodies to Hepatitis C.

**Fig 2:**
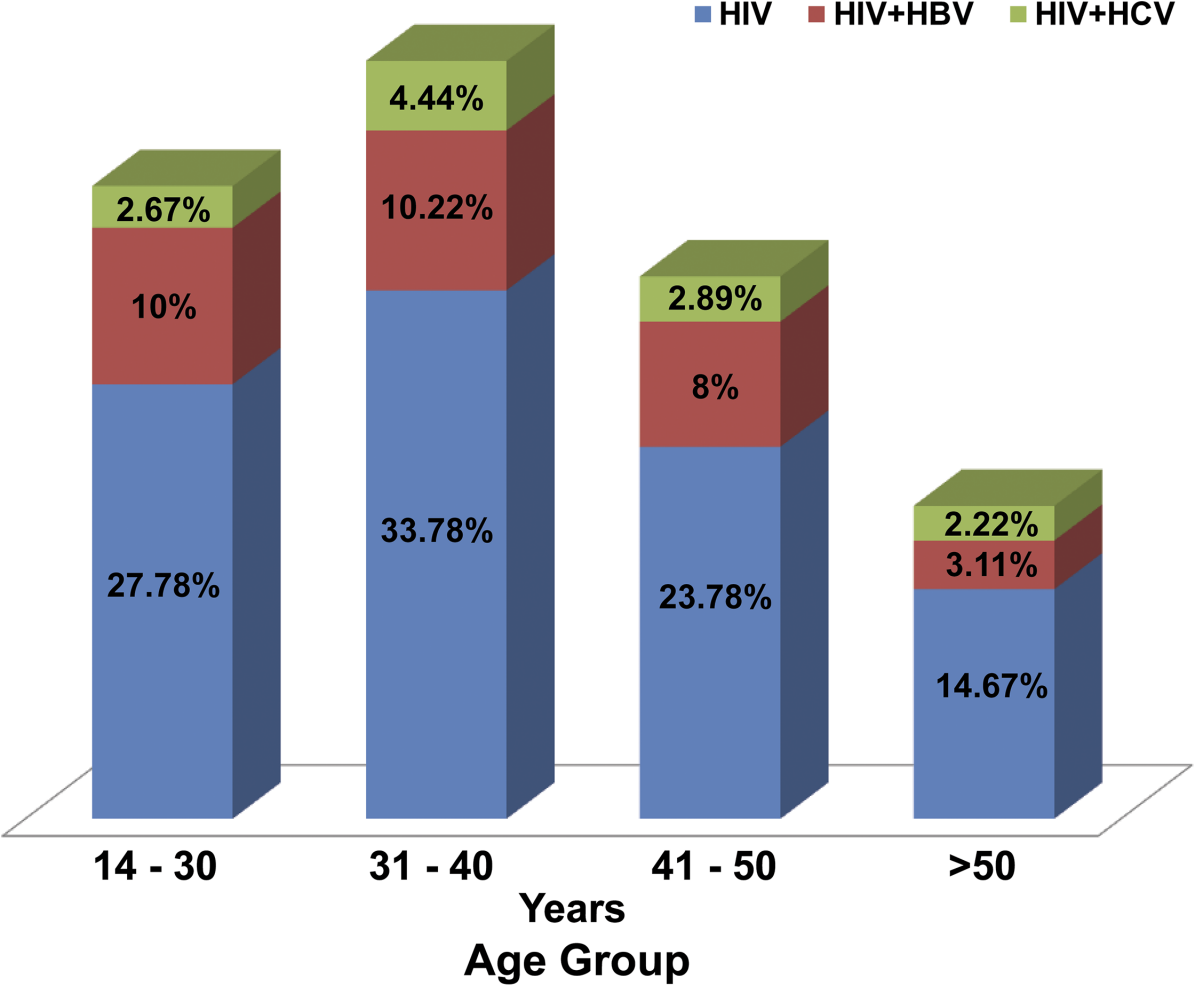
Age-specific prevalence for co-infection with HBV or HCV. Prevalence rates were calculated based on the proportion of study participants at risk of co-infection in a given age group.

Age specific prevalence for HBV was 10% for individuals 40 years old or younger, 8% for those of ages between 41–50 and 3% in subjects older than 50 years. Likewise HCV age specific prevalence was lowest (2%) in participants in the highest age group (>50 years) and highest (4%) in those with age 31–40 years (Fig 2).

**Fig 3:**
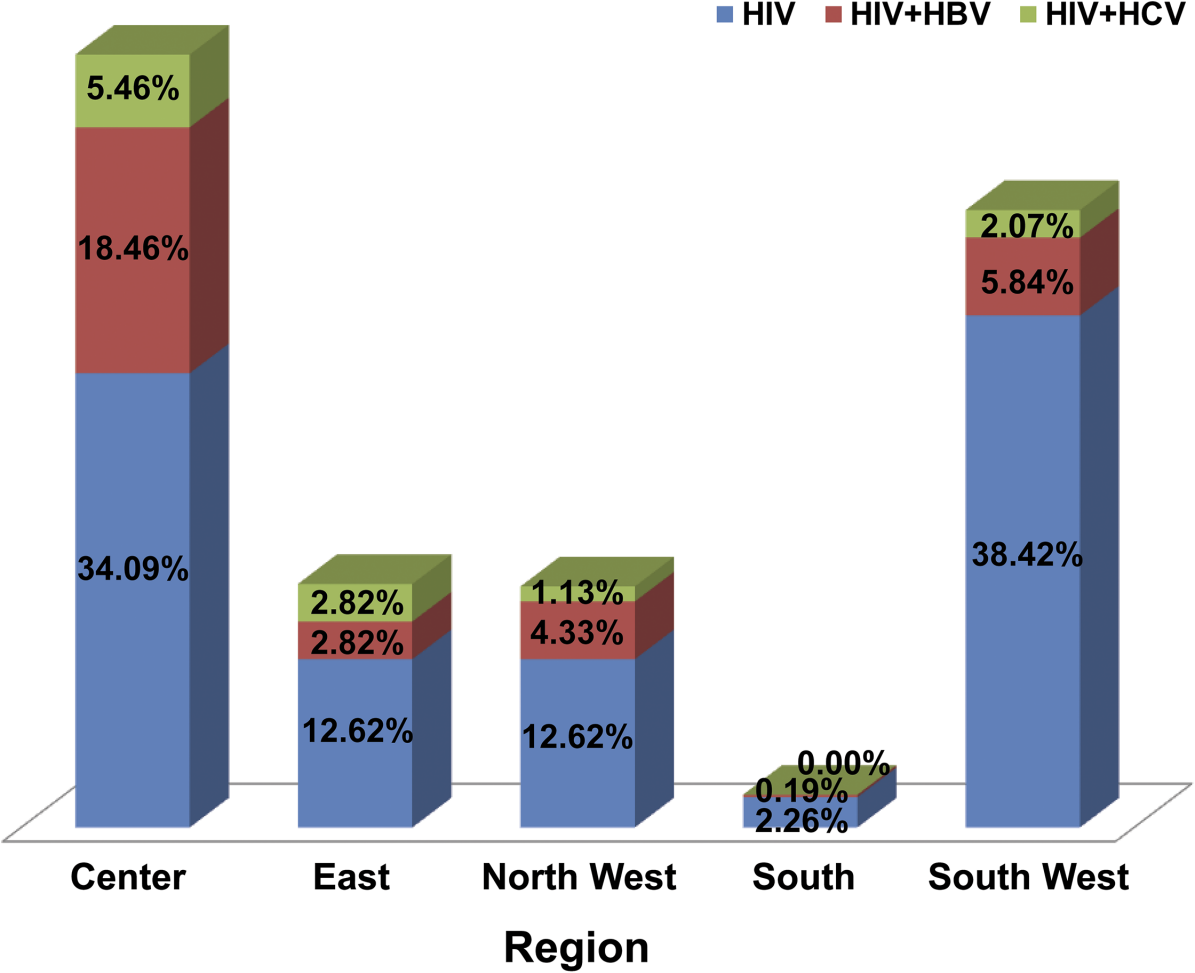
Region-specific prevalence for co-infection with HBV or HCV. Prevalence rates were calculated based on the proportion of study participants at risk of co-infection in a given region.

Analysis by region indicates that the Center Region had the highest HBV prevalence (18%) among the 181 HIV infected subjects studied (Fig 3). This is in contrast to the South West Region which had a similar number of HIV infected persons tested (n = 204) but a low HBV prevalence (6%), Fig 3. Co-infection with hepatitis C in the regions ranged from 0% for the South Region to 5% for the Center Region (Fig 3).

To determine the independent risk factors for co-infection with HBV or HCV in this HIV infected population, we performed an unconditional logistic regression with HBsAg positivity or HCV Ab positivity as outcomes adjusting for age, sex, CD4 count, residence, regions and number of sexual partners. Our results show that although age is not statistically associated with being chronically infected with HBV (HBsAg+), individuals older than 30 years were less likely to be infected compared to those with a younger age. These results also show that men had a higher chance of being infected with HBV than women, [OR = 2.39, (95%CI = 1.01–5.7)] (Table 3).

**Table 3:** Associations of demographic and serological markers with risk of infection with HBV or HCV.

Risk Factor	Hepatitis B surface antigen, N (%)				Hepatitis C antibodies, N (%)			
	*HBsAg+*	*HBsAg-*	*OR(95%CI)*	*Ptrend*	*Present*	*Absent*	*OR(95%CI)*	*Ptrend*
**Age Group**				**0381**				**0.582**
14–30	33(36.2)	92(22.7)	Reference		6(15.8)	119(24.1)	Reference	
31–40	38(30.2)	114(28.1)	0.69(0.25–1.9)		12(31.6)	140(28.4)	1.56(0.25–9.8)	
41–50	26(20.6)	81(20.0)	0.62(0.22–1.8)		8(21.0)	99(20.1)	0.67(0.08–5.3)	
>50	7(5.6)	59(14.6)	0.43(0.10–1.8)		8(21.0)	58(11.7)	1.70(0.19–14.5)	
**Sex**				**0.658**				**0.509**
Female	79(62.7)	240(59.3)	Reference		21(55.3)	298(60.4)	Reference	
Male	32(25.4)	118(29.1)	2.39(1.01–5.6)		13(34.2)	137(21.8)	0.40(0.05–1.8)	
**CD4 Count**				**0.425**				**0.481**
<250	16(20.3)	49(24.7)	Reference		3(16.7)	62(23.9)	Reference	
> = 250	63(79.7)	149(75.3)	0.37(0.15–0.9)		15(83.3)	197(76.1)	0.47 (0.10–2.4)	
Residence				**0.01**				**0.106**
Rural	14(11.1)	87(21.5)	Reference		11(28.9)	90(18.3)	Reference	
Urban	112(88.9)	318(78.5)	2.19(1.9–4.0)		27(71.1)	403(81.7)	0.58(0.26–1.1)	
**Regions**				**0.0001**				**0.013**
Western	40(31.7)	231(57.0)	Reference		12(31.6)	259(52.5)	Reference	
Eastern	86(68.3)	174(43.0)	3.20 (1.6–6.3)		26(68.4)	234(47.5)	2.16(0.70–6.7)	
**Sexual Partners**								**0.397**
One	41(72.9)	141(81.5)	Reference		14(87.5)	168(78.5)	Reference	
Multiple	16(28.1)	32(18.5)	1.28(0.60–2.7)		2(12.5)	46(21.5)	0.40(0.08–1.9)	

Percentages (%) in columns were calculated by dividing the number of participants with a particular outcome by the total number of participants with the outcome of interest *100. Adjustments were made for age, sex, CD4 count, residence, region and number of sexual partners.

HBV, Hepatitis B virus; HCV, Hepatitis C virus

We studied the effect of a lowering of CD4 level on co-infection in this HIV infected population by stratifying our study subjects into those with a CD4 count of below 250 cells/μl and those with CD4 counts ≥250 cells/μl. Our results indicate that subjects with a CD4 count superior to 250 cells/μl were less likely to be co-infected when compared to individuals with a lower CD4 count, [OR = 0.37, (95%CI = 0.15–0.9)] for HBV and [OR = 0.47, (95%CI = 0.10–2.4)] for HCV (Table 3).

We examined the influence of residence (rural village or urban city) on the chance of being co- infected with HBV or HCV. Our results show that participants living in urban cities were more likely to be chronically infected with HBV than those residing in rural villages, [OR = 2.19, (95%CI = 1.9–4.0), p = 0.01], Table 3. On the contrary, people living in cities were less likely to be infected with HCV compared to those in rural villages although the difference was not statistically significant, [OR = 0.58, (95%CI = 0.26–1.1), p = 0.10], Table 3.

We tested the hypothesis that participants with multiple sexual partners were more likely to be co-infected with either HBV or HCV compared to those with only one sexual partner. In support of our hypothesis, we observed a 28% increased chance of being infected with HBV in people with multiple sexual partners compared to those with only one, [OR = 1.28, (95%CI = 0.60–2.7)], Table 3. On the other hand the chance of being infected with HCV was higher in people having single sexual partners than in those reporting multiple partners [OR = 0.40, (95%CI = 0.08–1.9)], Table 3. In both cases, the results were not significant statistically.

Although we collected data from five political regions, for the purpose of this analysis and to maintain statistical power, we pooled data from the five political regions to make up two regions based on their geographical location, Western and Eastern regions. Compared to the western regions, participants from Eastern regions were statistically significantly at higher risk of being chronically infected with HBV, [OR = 3.2, (95%CI = 1.6–6.3), p = 0.0001], Table 3. Similar results were reported for infection with HCV [OR = 2.16, (95%CI = 0.7–6.7), p = 0.013], Table 3.

To better control for geographical location within the country, we performed separate analysis to determine which risk factors influence hepatitis virus co-infection in HIV infected people living in each of the two geographical settings, Western and Eastern parts of the country. The results are presented in Tables A and B in S1 File. These results indicate that age is a significant determinant of risk of infection with hepatitis B in Eastern part of Cameroon but not in the Western region. In this population, people older than 50 years had a significantly reduced chance of being infected with HBV [OR = 0.24, (95%CI = 0.06–0.9)].

## Discussion

Hundreds of thousands of people in Cameroon are infected with HIV with an estimated prevalence of 4.3% in calendar year 2011 [22] but little data are available on their HBV/HCV co-infection status despite recommendations from the WHO that these patients be placed on ART irrespective of their CD4 count. The purpose of this study was to determine the prevalence of hepatitis B or C infections in people living with HIV in some regions of Cameroon and to describe the associated risk factors. Overall 164 or 31% of the participants were co-infected with either HBV or HCV, with only about 1% co-infected with both viruses (Table 2). Prevalence of chronic HBV infection was 24% while that for chronic HCV was 7.2% (Table 2). Twelve percent of participants with HBV infection were also HBe-Ag positive. Age specific prevalence rates were lower than the general prevalence rates for both HBV and HCV (Fig 2). Risk factors significantly associated with HIV/HBV co-infection were being male, having a lower CD4 count, living in an urban setting and coming from the Eastern region of Cameroon (Table 3). For those living in the Eastern part of the country, age was also significantly associated with co-infection with HBV (Table B in S1 File)

The high frequency of co-infection with HBV in this population has important consequences for the emergence of liver-related mortality [23] and serious negative impact on the pathophysiology of HIV infection [24], including the possible doubling of the risk of death compared to individuals infected with HIV alone [25]. A direct benefit of knowing ones HIV/HBV or HIV/HCV co-infection status is in the initiation of ART. WHO recommends that HIV patients co-infected with HBV or HCV commence ART irrespective of their CD4 cell-counts. Unfortunately these guidelines are not adhered to because most HIV infected individuals don’t know their co-infection status. Instead these patients commence ART only after their CD4 cell counts have fallen below a certain level, typically below 350 cells/ul. Even then their ART regimens may not necessarily take into consideration the possibility of co-infection and therefore not include the recommended treatment for HBV or HCV. Our results confirm the need to scale up access to viral hepatitis screening in Cameroon to permit early diagnosis of co-infection in newly diagnosed HIV-patients to allow early initiation of the appropriate ART irrespective of CD4 cell counts. Knowing one’s co-infection status is also beneficial to those who are not yet infected with HBV as these individuals may be administered the hepatitis B vaccine thereby reducing their risk of co-infection with the virus.

The frequency of anti-HCV in this study (7.2%) is much lower than that reported previously in Cameroon [26] but much higher than reported from similar studies conducted in other Sub-Saharan African countries [27–30]. In HIV/HCV co-infected populations, persistent HCV-RNA is associated with an increased risk of cardiovascular and renal disease compared with HIV monoinfected patients [31–34]. Thus, although currently there is no vaccine for HCV, knowing one’s HCV status and commencing the recommended ART in a timely manner may help reduce the risk of developing these complications.

We examined the influence of several factors including, age, sex, CD4 cell count, residence, geographical region and number of sexual partners on the risk of co-infection with HBV or HCV (Table 3 and Figs 2 and 3). Our results indicate that age specific co-infection rates for HBV (10.2%) and HCV (4.4%) were highest in the age group 31 to 40 years. The relationship between age and infection with the hepatitis virus has previously been examined in several studies, with inconsistent results. Whereas some studies found a link between age and hepatitis B/C viral co-infection in HIV patients [35], others found no such association [36]. In settings like Cameroon where the prevalence of injection drug is very low, a high prevalence of viral co-infection may be attributable to a sexual mode of transmission as adults in this age group (30 to 40 years) are usually very sexually active.

Accepted values for CD4 cell counts in healthy individuals range from 500–1200 cells/μl [14]. HIV infection leads to a progressive reduction in the number of T cells expressing CD4. In the current study, the mean CD4 cell count was 400 cells/ μl (Table 2), well below the accepted minimum. Interestingly, we observed a significant association between having a lower CD4 cell count and risk of co-infection. Subjects with a higher CD4 count were less likely to be co-infected with HBV or HCV compared to patients with a lower CD4 cell count (Table 3). This association was significant for HBV but not for HCV probably due to a fewer number of patients with HCV infection. This result supports the biology of co-infection in that HIV infection is likely to result in a lowering of virus specific cell mediated immune responses thus paving the way for opportunistic infections including HBV and HCV to thrive and replicate [27–30]. CD4 cells, also known as helper T- cells (Th1/Th2) help fight infection by producing cytokines such as IFN-g (Th1) that generally promote cell-mediated immunity or IL-4 (Th2) which fights infection by triggering humoral immune responses through antibody production. It is therefore logical that those with a lower CD4 count should be at higher risk of co-infection. Our findings are supported by an international study that observed a significantly lower baseline CD4 T-cell count in HBV co-infected individuals compared with HIV-mono-infected before therapy [37]. However, the cross-sectional design of our study limits clear identification of cause-and-effect association between CD4 count and HBV and HCV infection. Thus, an alternative explanation to our observation could be that hepatitis B viral co-infections may have cause further damage to the patients’ immune system which can subsequently boost HIV replication and lower CD4 counts.

We found a significantly elevated risk of co-infection in residents of urban cities compared to those residing in rural villages. Although we did not examine the direct influence of socioeconomic status (SES) on risk of co-infection in this study, it has previously been reported that higher SES is associated with a more mobile lifestyle and a higher risk of infection with HIV [38]. Therefore, the higher rate of co-infection amongst urban dwellers in our study may be a reflection of their higher SES and greater mobility.

Although the proportion of women with chronic HBV or HCV infections (63%) is much higher than that for men (Table 1), in adjusted analysis (Table 3), being males was significantly associated with a higher risk of infection with HBV [OR = 2.4, (95%CI = 1.01–5.6)]. These results are consistent with published data from Cameroon [26]. The association of number of sexual partners with HIV/HBV/HCV infection was not significant. Although having multiple sexual partners has been associated with higher probability of co-infection with HBV [39] only a few people volunteered information of this parameter, hence our study lacked the power to find a significant association. A study in Nepal revealed that 33.4% of HBV/HIV co-infected individuals had history of having had multiple sex partners [40].

There are some limitations of this study that warrant further research. The analysis has been performed with samples from HIV-1 infected persons that were studied for HIV-1 prevalence and genetic diversity; thus, the samples were a convenience sampling set and were not collected randomly to represent the general population. A larger sample size representing the general HIV-1 infected population and/or a more complete data set will benefit more statistical power for some risk factors. The analysis performed here were all based on ELISA screening and no molecular tests were performed. We acknowledge that the use of additional HBV and HCV confirmatory tests as well as molecular tests for HBV DNA will also benefit the study. We note that in the absence of molecular tests for HBV DNA, it was not possible to take into account occult HBV infections in those with HBsAg negative results thereby possibly reporting a lower HBV prevalence than would otherwise have been the case. We also note that we did not perform test for HCV RNA and were therefore unable to identify active HCV infections among those who were anti-HCV positive. About 25% of HCV infections clear spontaneously resulting in a positive anti-HCV test but a negative HCV RNA PCR test [41]. Thus, we might have over reported a high prevalence of HCV infection. Despite these weaknesses, our study still reports findings of significant public health importance revealing a high frequency of co-infection with HBV in HIV infected individuals in Cameroon with risk factors for co-infection in this population that includes older age, having a lower CD4 cell count and living in urban cities. This study now opens new doors to begin to understand HIV-1/HBV and/or HCV co-infection in Cameroon over the past decade and to design more structured studies that would investigate co-infection in targeted populations in different geographic populations and risk groups. Importantly, this study suggests an urgent need to implement a systematic approach to the testing of all patients diagnosed with HIV for possible co-infection with hepatitis B or C. As such, those not yet infected with HBV can be given the hepatitis B vaccine while those already infected are immediately placed on the appropriate ART irrespective of their CD4 count.

## Supporting Information

S1 File Supporting Tables. Table A. Risk factors associated with infection with Hepatitis B or C in patients infected with HIV in the Western Regions of Cameroon, Table B. Risk factors associated with infection with hepatitis B or C in patients infected with HIV in Eastern regions of Cameroon.
(PDF)
