# Supplementary material for: Hepatitis B and C Co-Infections in Some HIV-Positive Populations in Cameroon, West Central Africa: Analysis of Samples Collected Over More Than a Decade
Source: PLoS One. 2015 Sep 15;10(9):e0137375. doi: 10.1371/journal.pone.0137375 (PMC4570762; doi:10.1371/journal.pone.0137375)
Supplement: S1 File — Table A. Risk factors associated with infection with Hepatitis B or C in patients infected with HIV in the Western Regions of Cameroon, Table B. Risk factors associated with infection with hepatitis B or C in patients infected with HIV in Eastern regions of Cameroon. (PDF) [file pone.0137375.s001.pdf]

Table A: Risk factors associated with infection with Hepatitis B or C in patients infected with HIV in the Western Regions of Cameroon

Risk Factor	Hepatitis B surface antigen, N (%)				Hepatitis C antibodies, N (%)			
	HBsAg+	*HBsAg-*	*OR(95%CI)*	*Ptrend*	*Present*	*Absent*	*OR(95%CI)*	*Ptrend*
**Age Group**				**0.457**				**0.473**
**14-30**	6(15.0)	31(13.4)	Reference		0(00)	37(14.2)	Reference	
**31-40**	9(22.5)	79(34.2)	0.59(0.19-1.8)		5(41.7)	83(32.0)	1.0(NA)	
**41-50**	9(22.5)	44(19.0)	1.00(0.34-3.3)		3(25.0)	50(19.3)	1.34(0.14-12.5)	
**>50**	4(10.0)	31(13.4)	0.67(0.17-2.3)		1(8.3)	34(13.1)	1.63(0.15-17.0)	
**Sex**				**0.740**				**0.334**
**Female**	25(62.5)	137(59.3)	Reference		8(66.7)	154(59.5)	Reference	
**Male**	8(20.0)	59(25.5)	2.57(0.49-13.4)		1(8.3)	66(25.5)	0.26(0.03-2.3)	
**CD4 Count**				**0.062**				**0.346**
**≤250**	10(25.0)	39(16.9)	Reference		1(25.0)	48(41.7)	Reference	
**>250**	6(15.0)	64(27.7)	0.22(0.05-0.9)		3(75.0)	67(58.3)	1.7(0.14-41.8)	
**Residence**				**0.044**				**0.349**
**Rural**	0(00)	18(7.8)	Reference		0(00)	18(6.9)	Reference	
**Urban**	40(100)	213(92.2)	1(NA)		12(100)	241(93.1)	1(NA)	

Associations of demographic and serological markers with risk of infection with HBV or HCV for subjects living in the Western Region (North West and South West) of Cameroon. Percentages for the infected and non-infected may not add up to 100 because some subjects have missing data on age and/or sex.

Table B: Risk factors associated with infection with hepatitis B or C in patients infected with HIV in Eastern regions of Cameroon

Risk Factor	Hepatitis B surface antigen, N (%)				Hepatitis C antibodies, N (%)			
	HBsAg+	*HBsAg-*	*OR(95%CI)*	*Ptrend*	*Present*	*Absent*	*OR(95%CI)*	*Ptrend*
**Age Group**				**0.009**				**0.118**
**14-30**	27(31.4)	61(35.1)	Reference		6(23.1)	82(35.0)	Reference	
**31-40**	29(33.7)	35(20.1)	1.87(0.95-2.6)		7(26.9)	57(24.4)	0.70(0.08-5.8)	
**41-50**	17(19.8)	37(21.3)	1.04(0.50-2.1)		5(19.2)	49(20.9)	0.76(0.09-6.2)	
**>50**	3(3.5)	28(16.1)	0.24(0.06-0.9)		7(26.9)	24(10.3)	3.44 (0.31-37.3)	
**Sex**				**0.547**				**0.141**
**Female**	54(62.8)	103(59.2)	Reference		13(50.0)	144(61.5)	Reference	
**Male**	24(27.9)	59(33.9)	(0.41-0.4)		12(46.0)	71(30.3)	0.75(0.07-7.3)	
**CD4 Count**				**0.838**				**0.797**
**≤250**	6(9.5)	10(10.5)	Reference		2(14.3)	14(9.7)	Reference	
**>250**	57(90.5)	85(89.5)	0.34(0.10-1.1)		12(85.3)	130(90.3)	0.25(0.04-1.7)	
**Residence**				**0.0001**				**0.259**
**Rural**	14(16.3)	69(39.7)	Reference		11(42.3)	72(30.8)	Reference	
**Urban**	72(83.7)	105(60.3)	3.70(1.89-7.2)		15(57.7)	162(69.2)	0.82(0.31-2.17)	

Associations of demographic and serological markers with risk of infection with HBV or HCV of subjects living in the Eastern Region (Eastern, South and Center) of Cameroon. Percentages for the infected and non-infected may not add up to 100 because some subjects have data missing on age and/or sex.
